# Extracellular Vesicles as Biomarkers of Brain Cellular Dynamics: Applications in Alzheimer's Dementia, Mild Cognitive Impairment and Late‐Life Depression

**DOI:** 10.1002/alz70856_103935

**Published:** 2025-12-24

**Authors:** Erica Vieira, Yuliya Nikolova, Sarah Elmi, Ana Paula Mendes‐Silva, Sanjeev Kumar, Breno S Diniz, Tarek K. Rajji

**Affiliations:** ^1^ CAMH ‐ Centre for Addiction and Mental Health, Toronto, ON, Canada; ^2^ University of Toronto, Toronto, ON, Canada; ^3^ Centre for Addiction and Mental Health, Toronto, ON, Canada; ^4^ Ontario Shores Centre for Mental Health Sciences, Whitby, ON, Canada; ^5^ University of Saskatchewan, Saskatoon, SK, Canada; ^6^ Temerty Faculty of Medicine, University of Toronto, Toronto, ON, Canada; ^7^ University of Connecticut Health Center, Frmington, CT, USA; ^8^ UT Southwestern Medical Center, Dallas, TX, USA

## Abstract

**Background:**

Exosomes from brain cells, such as neuron‐derived (NDEs) and astrocyte‐derived exosomes (ADEs), can cross the blood‐brain barrier and be detected in peripheral circulation. These vesicles facilitate communication between the central nervous system (CNS), neuroendocrine, and immune systems, carrying miRNAs and proteins that are protected from degradation. While exosomes are well‐studied in Alzheimer's Disease (AD), their role in Mild Cognitive Impairment (MCI) and Late‐Life Depression (LLD)—which may be prodromal stages of dementia—remains unclear. This project aims to identify biosignatures in NDEs, ADEs, and plasma to explore molecular profiles shared by LLD, MCI, and AD.

**Method:**

We recruited 40 LLD subjects, 25 with MCI, 24 with AD, and 31 age‐ and gender‐matched healthy controls. After psychiatric evaluation, blood samples were collected, centrifuged to obtain platelet‐free plasma, and stored at ‐80°C. Total exosomes were isolated using size exclusion chromatography and analyzed via NanoSight Pro nanotracking. NDEs and ADEs were quantified using the vFC™ vesicle flow cytometry kit on a CytoFlex system, while protein cargo was analyzed with Quanterix, SMCpro, and LX200. Five neuropsychiatric‐associated miRNAs were assessed: miR‐100‐5p, miR‐l‐3p, miR‐184, miR‐221‐3p, miR‐766, and miR‐5680.

**Result:**

LLD and MCI showed no differences in NDEs and ADEs compared to controls, while AD had elevated levels. No microRNA differences were found between LLD and controls, but five of six miRNAs were reduced in AD. Proteomic analysis revealed persistent neuroinflammation in AD compared to LLD and MCI.

**Conclusion:**

These findings suggest compromised brain‐periphery communication in MCI, LLD, and AD, highlighting exosomes as a window into the molecular pathology of these disorders.

